# Signal-based differentiation of essential and dystonic tremor

**DOI:** 10.3389/fneur.2026.1910178

**Published:** 2026-09-07

**Authors:** Veronika Selzam, Verena Häring, Juan Francisco Martin-Rodriguez, Petra Schwingenschuh, Pablo Mir, Rick C. Helmich, Kailash P. Bhatia, Jens Volkmann, Robert Peach, Sebastian R. Schreglmann

**Affiliations:** 1 Department of Neurology, University Hospital Würzburg, Würzburg, Germany; 2 Unidad de Trastornos del Movimiento, Servicio de Neurologia y Neurofisiologia Clinica, Instituto de Biomedicina de Sevilla, Hospital Universitario Virgen del Rocio / CSIC / Universidad de Sevilla, Seville, Spain; 3 Centro de Investigación Biomédica en Red sobre Enfermedades Neurodegenerativas (CIBERNED), Instituto de Salud Carlos III, Madrid, Spain; 4 Departamento de Psicología Experimental, Facultad de Psicología, Universidad de Sevilla, Seville, Spain; 5 Department of Neurology, Medical University of Graz, Graz, Austria; 6 Departamento de Medicina, Facultad de Medicina, Universidad de Sevilla, Seville, Spain; 7 Department of Neurology, Donders Institute for Brain, Cognition and Behavior, Radboud University Medical Center, Nijmegen, Netherlands; 8 Department of Clinical and Movement Neurosciences, UCL Queen Square Institute of Neurology, London, United Kingdom; 9 Department of Brain Sciences, Imperial College London, London, United Kingdom

**Keywords:** big data, dystonic tremor, essential tremor, machine learning, tremor analysis

## Abstract

**Background:**

There is substantial overlap in the perceived clinical presentation of essential tremor (ET) and dystonic tremor (DT). As both etiologies require different treatment approaches, establishing the correct clinical diagnosis remains key. Massive time series feature extraction and machine learning allows the examination of oscillating biological signals in an objective manner.

**Objective:**

This study compares the spectrum of tremor characteristics in ET and DT patients across several academic centers to establish a generalizable differentiation.

**Methods:**

278 accelerometer recordings from a multi-center cohort of 78 patients (37 ET, 27 DT, 14 TaD), recorded according to center-specific study protocols, were combined into a single data set. Standard tremor characteristics were compared with clinical diagnosis serving as the gold standard. Massive higher-order feature extraction was consistently applied to tri-axial postural accelerometer recordings, followed by supervised and unsupervised statistical learning.

**Results:**

Raw time-series signals were analyzed for both standard characteristics and >7,000 extracted features. Although features reached an optimal differentiation accuracy up to 90.2% in individual centers, none generalized across cohorts, indicating large differences in clinical phenotyping. Unsupervised machine learning detected two distinct signal clusters.

**Conclusion:**

This study shows that ET and DT are diagnosed inconsistently between even expert centers, reflecting inclusion bias and the variability of clinical features influencing clinical phenotyping. Although un-biased, data-driven approaches cannot replace clinical diagnostic criteria, they can help to identify truly generalizable movement patterns in so far inconsistently diagnosed disorders. Replicable and consistent patient stratification will likely improve research into the pathophysiology and treatment of tremor.

## 1 Introduction

Tremor is defined as an involuntary rhythmic, sinusoidal oscillation in a body part, and tremor disorders affecting the upper limb/s is amongst the most common neurological disorders worldwide (1). Tremor can occur as one of several symptoms of a disorder or in isolation and is diagnosed clinically. Phenotypical differentiation between distinct tremor syndromes is based on characteristics such as frequency, distribution, effect of posture and the presence or absence of additional symptoms and signs (2), which can be enhanced by electrophysiology (3). Based on improved phenotypical observation and evolving concepts of disease entities, diagnostic categories and criteria are evolving over time (4–8). In parallel, knowledge of the underlying pathophysiology has become more granular (9–11) and accelerometric tremor quantification is much more widely used in research and clinical practice (12, 13).

Essential Tremor (ET) is clinically defined as an isolated, symmetrical, postural and kinetic tremor, mostly affecting the upper limbs over at least3 years—the latest diagnostic classification introduced the term ET syndrome to acknowledge the likely existence of several entities within ET (7). Action tremor however frequently also occurs in the context of dystonia (14–16). Dystonic tremor (DT) is defined as tremor in a body part that is affected by dystonia (14, 17, 18), while tremor associated with Dystonia (TaD) describes when tremor and dystonia are present in different body parts. While ET classically is referred to as regular and sinusoidal in nature, and DT more irregular, coarse and asymmetrical (3, 19), in clinical reality there is substantial overlap (20, 21). In effect, diagnostic uncertainty and differing diagnostic stratification persists even amongst scholars (22–24), despite differing evidence-based treatment suggestions for ET (25, 26) and DT (27). The subsequent lack of unique biomarkers continues to stir the debate on the most suitable and feasible tremor classification and terminology (8, 28).

Machine-learning offers the potential to identify generalizable patterns in tremor if applied vigorously in adequately powered, multi-center-datasets (29, 30). Unbiased, feature-based tremor stratification (31), based on massive time series feature extraction (32), is a powerful tool to stratify tremor disorders according to discrete signal patterns (30), or predict the response to therapeutic interventions (31).

We applied both standard tremor characteristics, as well as supervised machine learning of tremor time-series using feature extraction to a large, multi-center ET/DT accelerometry data set. In order to characterize generalizable differences, we utilized an unsupervised, purely-data driven clustering approach and identified discrete tremor signal clusters. This might serve as a basis to help define universally applicable diagnostic criteria to differentiate ET from DT.

## 2 Materials and methods

### 2.1 Participants and ethics approval

We included 278 accelerometer recordings of 78 individuals with hand action tremor clinically diagnosed as ET, DT or TaD from three centers (University Hospital Graz; Virgen del Rocío University Hospital, Seville; Institute of Neurology Queen Square London). In addition to routine neurological examination, standardized clinical rating scales for tremor were recorded (Supplementary Table 1). All patients were diagnosed by local specialists based on at least two independent consultations according to published criteria (6, 7). Some recordings had been included in previous publications (31, 33–35). All participants provided written informed consent prior to inclusion according to local ethics at each participating center. The study was approved by the local research ethics committee (Ethikkomission der Universität Würzburg Nr. 20210209 03) in accordance with the declaration of Helsinki.

### 2.2 Accelerometer examination

Accelerometer recordings were performed after withdrawal of oral anti-tremor medication (overnight) and botulinum-toxin injections (at least 3 months). Patients were seated comfortably in a chair and postural tremor recordings taken with both arms outstretched in front of them. Each accelerometer was firmly attached to the dorsum of the hand using center-specific settings and equipment (Supplementary Table 1). Patients with enhanced physiological tremor were excluded (12, 36).

### 2.3 Data analysis

#### 2.3.1 Data preparation

All analyses were performed in Matlab (R2021b; Mathworks, USA). For each recording the vector sum of triaxial accelerometer recordings was computed, cut to 10 s segment length, filtered using a second order Butterworth filter between 2 and 30 Hz, down sampled to 100 Hz and normalized with a mean of 0 and a standard deviation 1. All time-series raw data were checked for artifacts in raw format, frequency [Fast Fourier Transform (FFT)], and joint time-frequency domain (Wavelet Transform). For standardization and to increase comparability, for each patient with bilateral recordings, the more affected hand (30) was used consistently throughout.

#### 2.3.2 Standard tremor characteristics

To compare tremor disorders within and across centers, standard tremor characteristics (Supplementary Table 2) were calculated.

#### 2.3.3 Supervised machine learning

To calculate the classification accuracy of features a pipeline was constructed in MATLAB 2021b. To provide a meaningful basis for machine learning, all timeseries features were calculated for all *n* = 254 ET/DT postural tremor recordings using HCTSA (highly comparative time-series analysis) (32, 37), resulting in a 254 x 6387 matrix, and selected based on their Random Forest univariate classification accuracy per center (30). To create a balanced across-center normalization, one set of HCTSA features per patient was normalized with a mean of 0 and a standard deviation of 1. Afterwards, the extracted features of all recordings per patient were normalized using the centering and scaling values from the across-center normalization. MATLABs Machine Learning Algorithms TreeBagger, kNN, linear SVM, Neural Net and Linear Regression were applied with a leave-one-out cross-validation (LOOCV), ensuring all recordings from one patient were placed in the same fold. Bootstrapping of time-series was used to account for unbalanced data sets in the train set. Afterwards the best 50 features per center were identified according to their univariate classification accuracies. Features containing the same information were eliminated based on pairwise linear correlation. The 8 best uncorrelated features were sorted by univariate classification accuracies and used in descending combination (1&2, 1&2&3, ...) to detect the best performing feature combination.

#### 2.3.4 Unsupervised machine-learning

**Figure 1:**
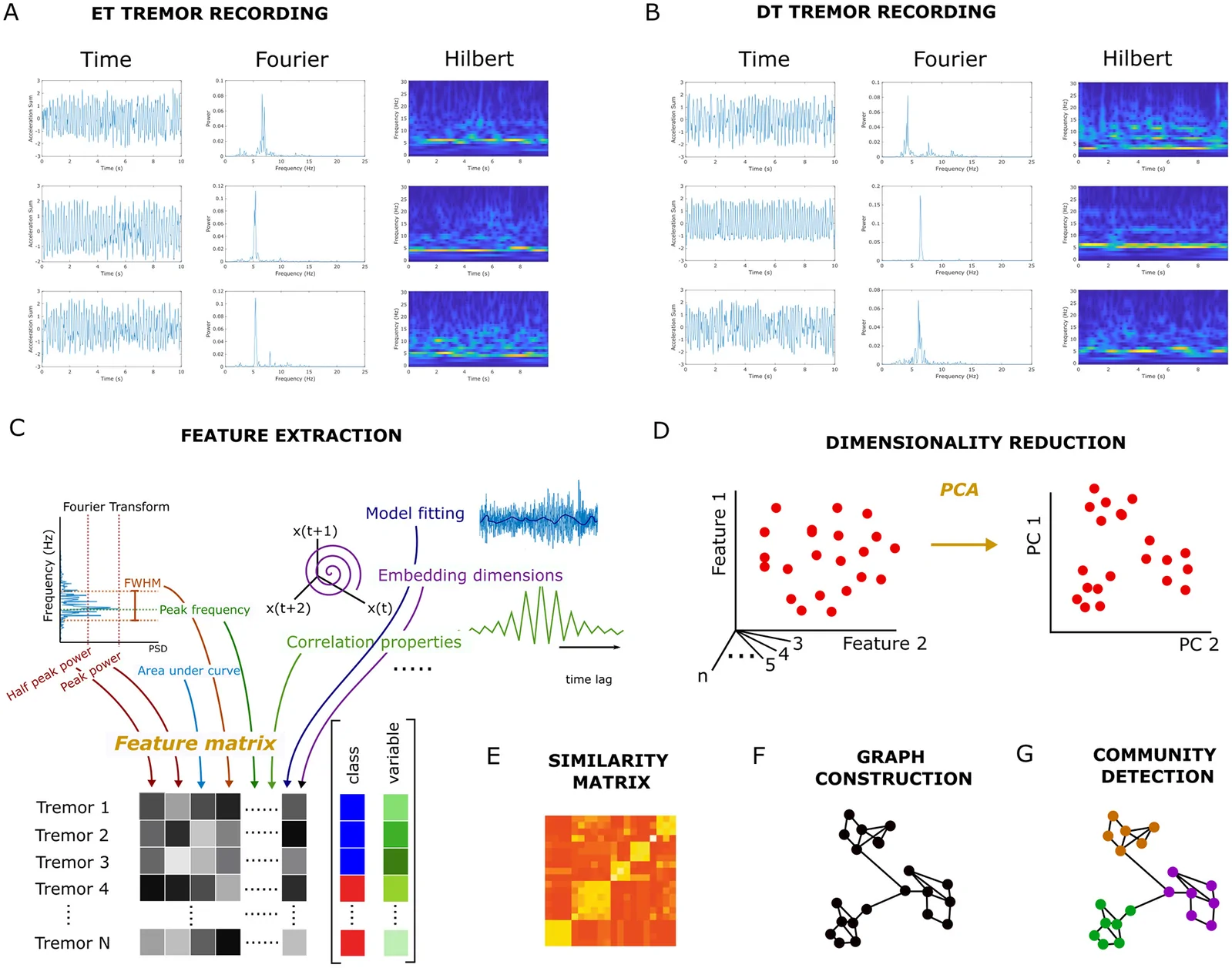
Workflow unsupervised machine learning and graph-based clustering analysis. First ET **(A)** and DT **(B)** tremors were recorded using a triaxial accelerometer, then data was prepared and analysed in time and frequency domain. Features were extracted **(C)**, dimensionality reduced **(D)** using PCA resulting in embedded features by the first 50 PCs **(E)**. Finally, a graph was constructed **(F)** and community detection was applied **(G)**.

Only one postural recording was used per patient for the workflow (Figure 1), performed in Python 3.9.7: all available HCTSA Features were normalized with a mean of 0 and a standard deviation of 1 per center to minimize the batch effects between institutes. Principal Component Analysis (PCA) with 50 PCs (Principal Components) was used for dimensionality reduction. Afterward a k-nearest neighbor graph was constructed using the cknn package (38) with patients as nodes and edges as similarities between feature values. To control for an even spread of data points from all centers across the graph, the variance to distributions in a graph $var=\frac{1}{2}\;\underset{i,\;j\;\in \mathbb{N}}{\sum }p\left(i\right)p\left(j\right)d^{2}\left(i,j\right)$ was computed (39). Markov Stability Algorithm was applied to calculate different partitions of the resulting patient nodes (40) with advancing Markov Time. To detect partition stability (38), the optimal scale was calculated using the pygenstability package (41).

To aid clinical interpretation, recordings from the resulting partitions were inspected and compared visually (FFT and Wavelet Transform), as well as for their standard tremor characteristics. In addition, HCTSA features were computed per cluster and supervised machine learning applied to detect and interpret features that could differentiate clusters.

### 2.4 Statistical analysis

Clinical and electrophysiological data were compared using MATLABs n-way ANOVA and Wilcoxon rank sum test. All tests were two tailed, the α level was set at *p* < 0.05.

### 2.5 Data availability

Raw data is available from individual contributing centers (according to data sharing arrangements governed by patient consent) by reasonable request to the corresponding author.

## 3 Results

### 3.1 Patient cohorts

Accelerometer recordings from 37 ET and 27 DT patients were included, revealing comparable age, sex, handedness, disease onset and disease duration - statistical analysis detected no difference within diseases per center and across centers (Supplementary Table 3). In addition, available 14 TaD patients from one center (Graz) were compared with ET and DT patients from the same center (Supplementary Table 4).

### 3.2 Neither standard characteristics nor feature based supervised machine learning generalize across centers

**Figure 2:**
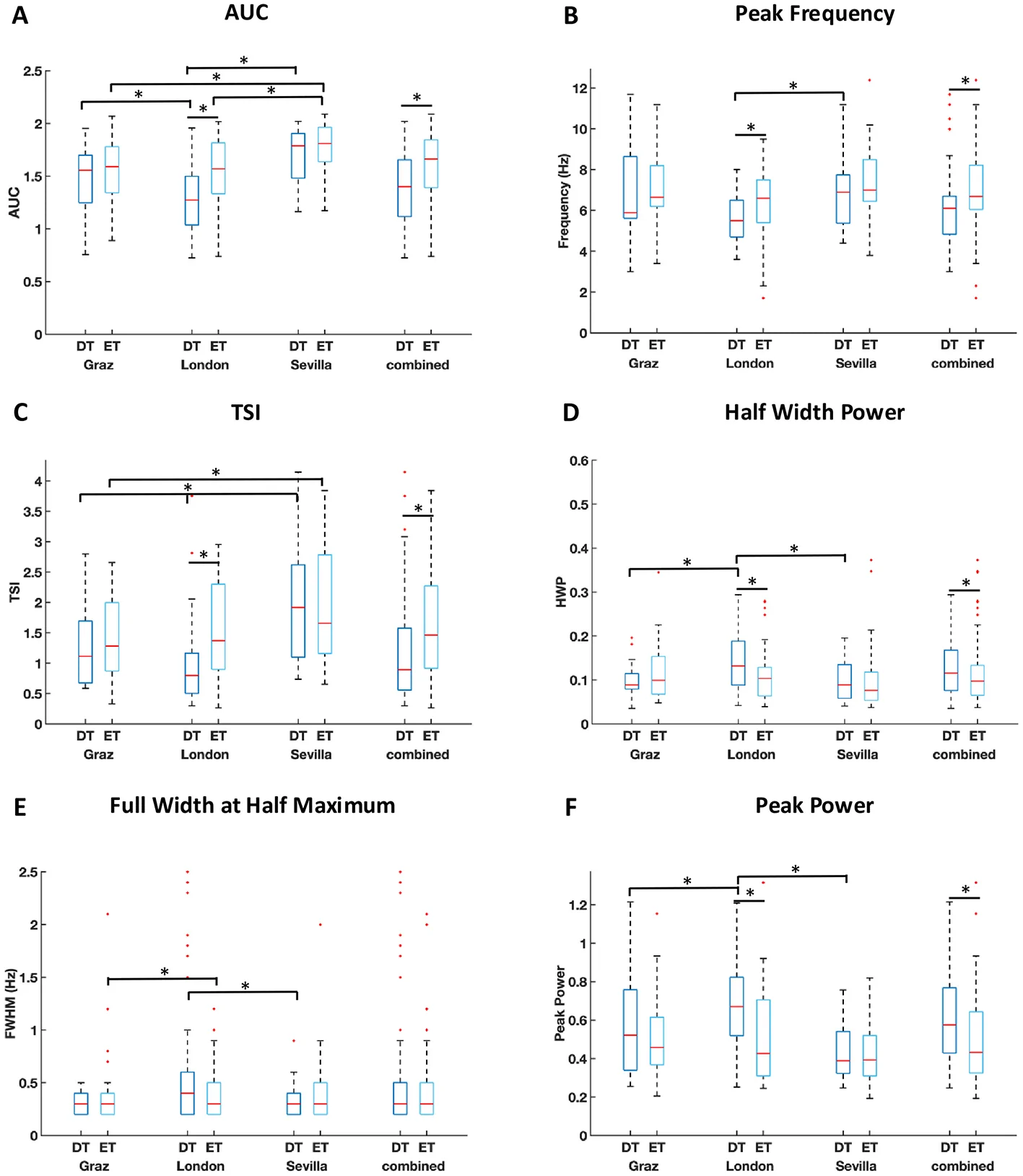
Standard Tremor Characteristics by and across centers. Distribution of AUC **(A)**, Peak Frequency **(B)**, TSI **(C)**, Half Width Power **(D)**, Full Width at Half Maximum **(E)** and Peak Power **(F)** visualized using boxplots per center and by diseases for DT and ET. AUC, Peak Frequency, TSI, HWP and Peak Power showed statistical differences between diseases and between centers. For FWHM statistical differences between centers but not between diseases were detected. * < 0.05.

We first assessed standard tremor characteristics per and across centers according to clinical diagnosis (Figure 2). AUC, peak frequency, TSI, HWP and peak power showed significant differences between diseases and across centers, whereas for FWHM only statistical differences between centers could be detected. However, none showed consistent differentiation ability between diseases for each center, with all features even presenting significant differences between centers within the same diagnosis. This suggests substantial differences in clinical phenotyping between centers.

We next explored in how far supervised feature-based machine-learning could identify an individual feature or a set of features differentiating reliably between ET and DT. In a first step, the univariate classification accuracy was computed for each center to detect the best performing individual features, reaching 87.2% for Graz, 71.4% for London and 78.3% for Sevilla. The same analysis comparing diagnoses across centers resulted in a substantially lower accuracy of 69.0% (Supplementary Table 5).

To increase classification accuracy, combinations of the best performing uncorrelated features were identified and compared. This resulted in a maximum classification accuracy of 90.2% for Graz, 71.7% for London and 78.3% for Sevilla based on various algorithms and combinations of up to 8 features. Again, applying the same methods to recordings from across all centers resulted in an unchanged classification accuracy of 69.0% (Supplementary Table 6). Overall, hence, utilizing feature combinations did not improve results over using individual best features. While differentiation was possible with fair to excellent classification accuracy in individual centers, feature-based machine learning was not able to identify generalizable patterns across centers.

### 3.3 Identifying patterns through feature-based learning

**Figure 3:**
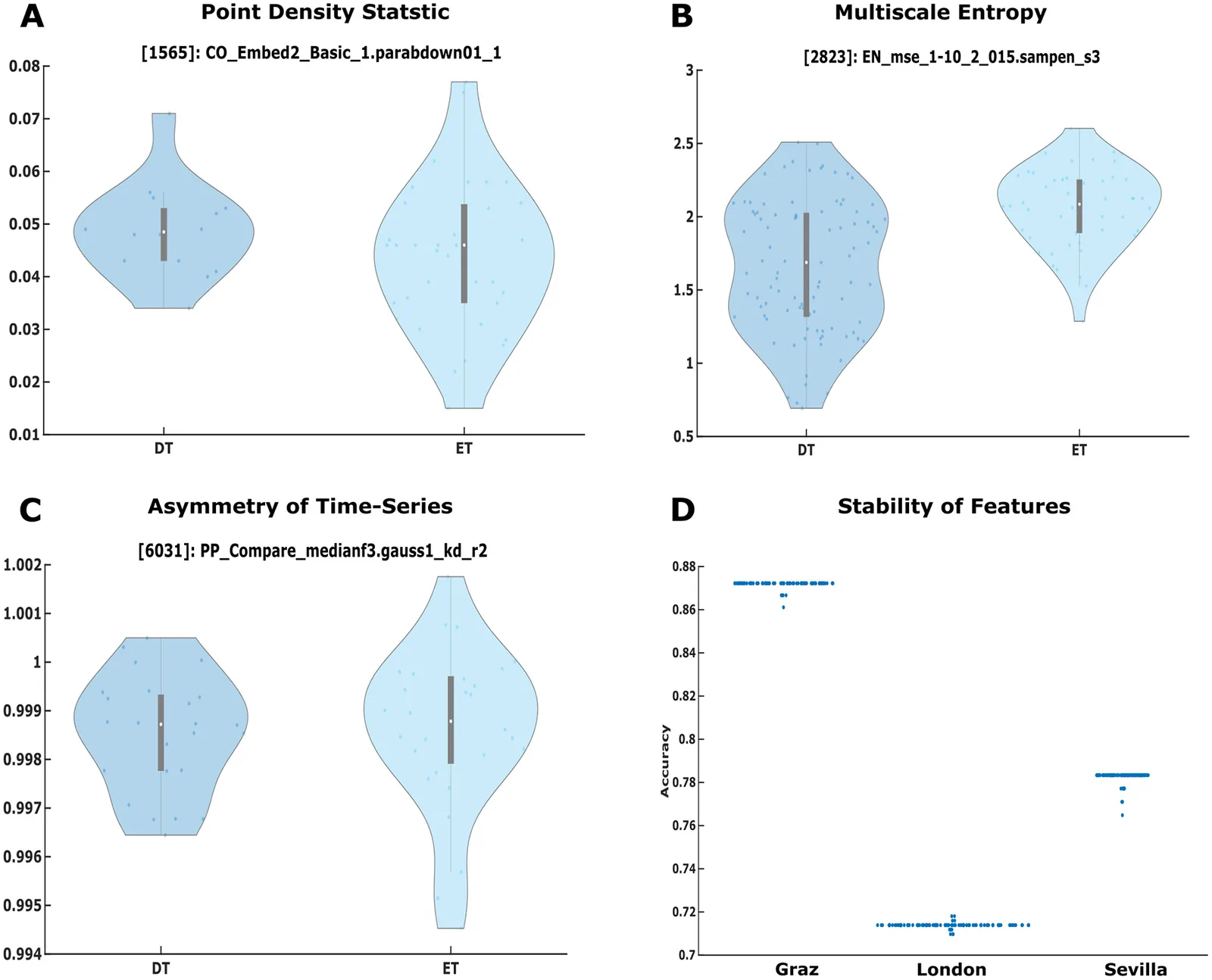
Distribution of feature values for best performing feature per center and the stability of the 8 best uncorrelated features per institute. The distribution of data points per feature and center suggests that tremor accelerometer recordings from **(A)** Graz and **(C)** Sevilla differed more between conditions than from **(B)** London. The displayed features (HCTSA feature name given next to feature number in brackets) are the best performing features per center using bootstrapping and LOOCV. Repeating the classification 100 times of the best feature per center shows the stability of these features for Graz, London and Sevilla **(D)**.

The interpretation of signal features can be used to detect patterns in accelerometer data (30, 31). We hence used the best individual features per center from above to explore underlying signal patterns. Based on the low accuracy of the combination across centers, we focused on the best performing feature per center (Figure 3).

From Graz, feature [1565] (CO_Embed2_Basic_1.parabdown091_1; Figure 3A) calculates a point density statistic from every time-series x using

$$\begin{array}{l} \frac{\sum |x_{t+1}+\left(x_{t}^{2}+1\right)|<0.1}{length\;x-1} \end{array}$$

Mathematically, this describes/quantifies periods, where the tremor intensity or pattern is relatively stable or showing minimal variation.

From London, feature [2823] (EN_mse_1-10_2_015.sampen_s3; Figure 3B) calculates the multiscale entropy of times-series. Entropy is a measure of the unpredictability or complexity of data. Multiscale entropy extends this concept by analysing the data at multiple scales. Multiscale entropy relates to more erratic or unpredictable, respectively more regular and predictable tremor patterns.

From Sevilla, feature [6031] (PP_compare_medianft3.gauss1_kd_r2; Figure 3C) detects the asymmetry of timeseries by using the ratio of *R*^2^ of the probability density estimate based on a normal kernel function gaussian fit on the third order median filtered time -series and the raw time-series. Asymmetry here refers to how much the distribution of data points deviates from a symmetrical pattern. All three identified features proved stable on repeat testing (Figure 3D). Taken together, while our feature-based stratification attempts did not identify generalizable features, feature-based supervised learning identified signal variability, symmetry and predictability as key signal characteristics to differentiate ET from DT tremor.

### 3.4 Comparison between DT and TaD

From one center additional TaD recordings were compared with DT and ET: apart from a female-dominance in the TaD group there were no relevant demographic differences between the groups (Supplementary Table 4).

AUC, TSI and Peak Power showed statistical differences between DT and TaD (Supplementary Figure 1). The feature value distributions between tremor diagnoses, as computed by the HCTSA Interface, similarly show that TaD and DT differ consistently across features (not shown). Accordingly, when lumping TaD and DT together, the maximum univariate classification accuracy to differentiate DT from ET in the Graz dataset decreased from 90.2% to 81.0%. This further supports the impression that TaD is distinct from DT.

### 3.5 Unsupervised Learning identifies two distinct tremor clusters

As the above analyses did not yield a generalizable signal pattern among clinically diagnosed tremor cases, we applied a purely data-driven approach, using unsupervised learning without diagnoses, to search for discrete, consistent and generalizable signal clusters among ET and DT recordings.

**Figure 4:**
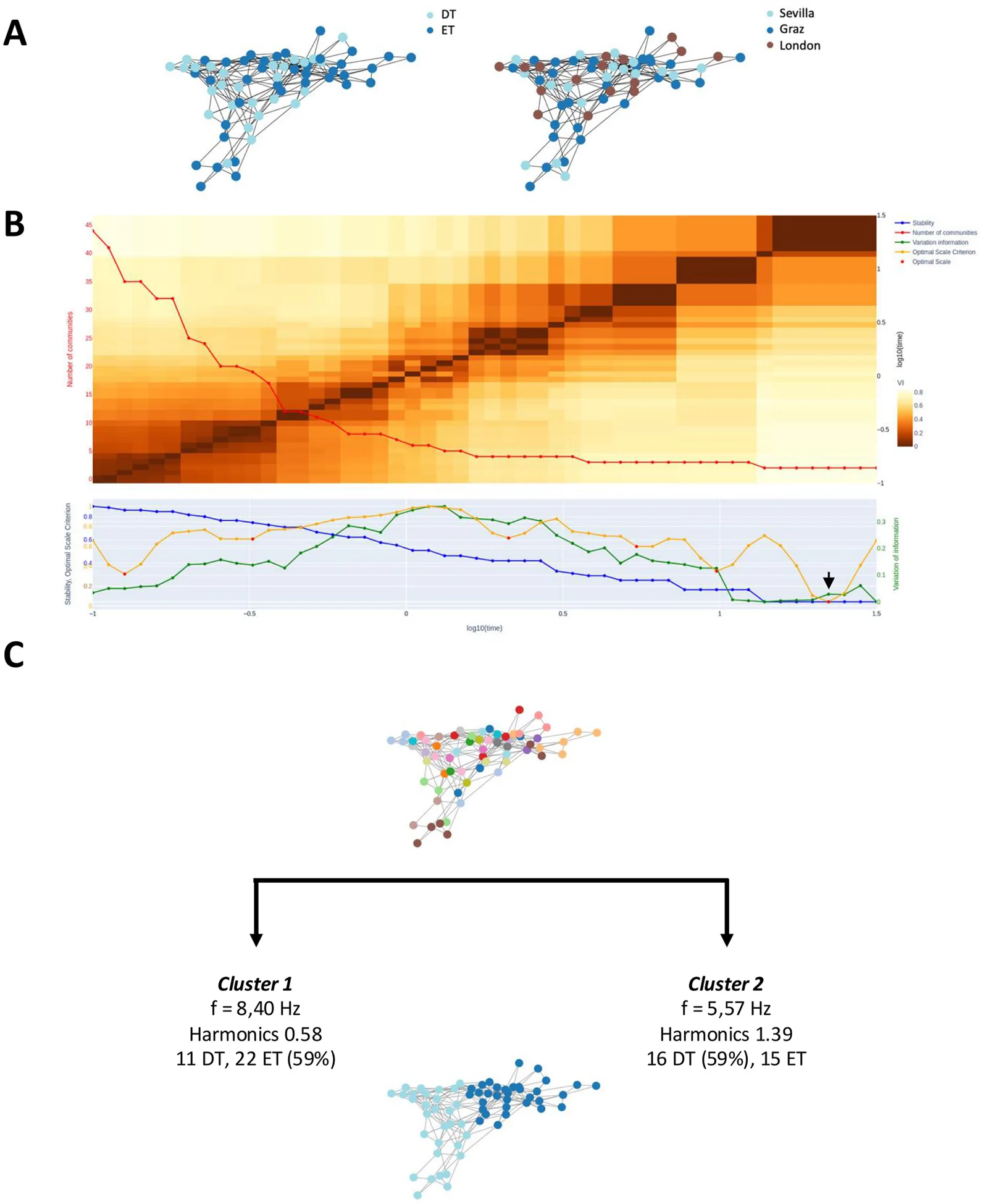
Multi-center unsupervised learning identifies clusters of homogeneous tremor across centers. **(A)** Recordings across conditions (ET and DT) and centers were combined in the same graph-based structure. Recordings from all centers show an even distribution across the common feature space **(B)** Markov stability analysis, based on the stability and optional scale criterion based on all HCTSA features, identified stable partitions with 2 clusters (arrowhead). **(C)** cluster and corresponding sub-cluster. With clinical diagnosis as reference the accuracy was 59% for ET and 59% for DT.

In a first step, a common feature space for all included recordings was established (Figure 4A). Calculating the variance to graph distributions resulted in a variance of 1.348 for Graz, 1.306 for Sevilla and 1.301 for London. This implies that data points for each center were distributed evenly across the common feature space, indicating an even representation of tremor signals. Next, Markov Stability analysis provided several mathematically possible partitions of the data based on stability and optional scale criterion (Figure 4B). The most stable partition at Markov Time *t* = 46 resulted in two distinct cluster:

Cluster 1 contains more clinical ET and Cluster 2 more clinical DT patients, resulting in a classification accuracy of 59% for each diagnosis. Standard tremor characteristics were found to differ significantly between resulting clusters (Table 1), with a higher Peak Frequency and lower HWP in Cluster 1, and higher peak power and higher number of harmonics in Cluster 2.

**Table 1:** Tremor-characteristics (clustering DT vs ET, Figure 4) the two cluster show statistical differences in Peak Frequency, HWP, Peak Power and Harmonics.

Characteristic	Cluster 1 (mean)	Cluster 2 (mean)	Comparison
Peak frequency	8.40 Hz	5.57 Hz	*P* = 3.8^*^10(^−7)
HWP	0.10	0.15	*P* = 6.7^*^10(^−4)
FWHM	0.37	0.54	*P* = 0.13
Peak power	0.43	0.63	*P* = 6.1^*^10(^−4)
Number of harmonics	0.58	1.39	*P* = 1.06^*^10(^−4)
AUC	1.56	1.59	*P* = 0.57
TSI	1.49	3.04	*P* = 0.13

To further understand the signal characteristics within clusters, we performed supervised machine learning using Clusters 1 and 2 as distinct labels and interpreted the 3 best performing least correlated features: The feature EN_SampEN_5_02.sampen5 (Cluster differentiation accuracy: 91,5%) calculates the sample entropy of time-series by evaluating the frequency and predictability of patterns of 5 consecutive data points within time-series–Cluster 1 has a lower feature value than Cluster 2, indicating a more regular and predictable pattern. The second feature SB_MotifThree_quantile.cbc (90,1%) uses a coarse-graining method to a 3-letter alphabet, which reduces the complexity of the small scale signal characteristics to assess signal variability– Cluster 1 has relative lower values than Cluster 2, pointing at a more regular pattern in cluster 1. The third feature SP_Summaries_welch_rect.statav2_s (90, 1%) calculates the variance across a broad frequency band which captures harmonics to the tremor signal‘s fundamental frequency– Cluster 1 has higher feature values, suggesting less harmonics.

All in all, these three features indicate that tremor signals in Cluster 1, which contains more ET, have a more similar, regular and predictable pattern and Cluster 2, which contains more DT, is more irregular and unpredictable.

## 4 Discussion

This study provides evidence that according to clinical diagnostic practice there is currently no uniformly identifiable signal marker to reliably differentiate ET and DT. Our results not only identify the limits of current diagnostic criteria and their interpretation, but may provide an un-biased, data-based groundwork for the development of future diagnostic criteria.

### 4.1 The inconsistent status quo– a diagnostic dilemma

This is the first sensor-based multi-center analysis of the ET/DT tremor spectrum. There are four previous sensor-based studies (20, 42–44) on single-center cohorts, while previous studies evaluating tremor in dystonia from several centers/with external validation were based on clinical observation (16) or hand-drawn spirals (45).

Our analyses provide a comprehensive accelerometric comparison of the current differentiation practice between ET and DT. Rigorous data harmonization, established in a previous multi-center accelerometer study (30), and the inclusion of standard characteristics, showing obvious inconsistencies between centers, argue against differences in data acquisition but clinical classification/phenotyping as the reason for our observations. Based on clinical phenotyping we could not identify any predefined or novel characteristic to reliably differentiate between the two entities, which confirms that this particular differentiation is– even amongst expert raters—highly subjective (16, 22). Although there was reasonable diagnostic consistency within each center, no signal feature universally generalized across centers. The analysis of feature value distributions suggests that in certain cohorts the difference between ET and DT was apparently more obvious, whereas in others, this was more similar, making the differentiation even harder. Although impossible to quantify, we acknowledge that referral, ascertainment and selection bias most certainly contributed to this effect, as none of the included cohorts stem from a representative population sample. It is similarly conceivable that cases seen at a tertiary referral center include less classic/distinct cases.

Like previous studies relying on accelerometer (30) or clinical data (16), the heterogeneity found between centers documents the strength of multi-center datasets, and at the same time greatly limits the validity of single center observations. Our results imply that current clinical diagnostic definitions of ET and DT cannot be applied in a consistent manner, and hence call for an improved diagnostic framework for ET/DT tremor differentiation.

### 4.2 Dystonic tremor–a discrete separate entity, but what is it?

According to the literature, tremulous movements are present in variable proportions of dystonia patients, ranging from 17% (46), 44% (47) and 48% (16) to 55% (48). There is a spectrum in involuntary movements from regular to irregular tremor to myoclonus (3, 49), and we agree that the term tremor should be reserved for rhythmic movements only (8). The large differences in tremor rates between studies (46, 48) and cohorts within studies (16) document the apparent difficulties and ambiguities in describing this frequent movement pattern. Cervical dystonia is the dystonia subtype with the highest tremor incidence, but more importantly, involved clinicians had a significant influence on the reported presence of tremor (16)—in line with our own findings, this documents the subjectivity of clinical phenotyping.

The non-supervised machine learning approach shows two reproducible clusters in our multi-center data set, equalling the number of clinical tremor categories included. Hence, clinical impression and multi-dimensional mathematical analysis confirm the presence of two distinct tremor entities. It is not surprising that the concordance rate between clinical and mathematical stratification is however poor, as differentiation based on best clinical phenotyping had proved not to generalize across centers.

Nevertheless, our approach allows the phenotypical characterization of two clusters, with mean frequency, sample entropy, number of harmonics and rhythmicity/regularity/predictability proving most relevant. Notably, these characteristics have previously been described as typical for ET (10), respectively DT (20).

Historically, the term DT was coined by Stanley Fahn (50), as many of the patients referred to him with a diagnosis of ET showed dystonic features (51). His reasoning was probably influenced by a previous Japanese EMG study, which had reported an irregular tremor in patients with dystonia (52). Fahn‘s differentiation was based purely on clinical description, and focussed more on phenomenology, i.e., activation and postural patterns, and less on rhythmicity, whereas over time tremor regularity and jerkiness increasingly became defining characteristics of DT (28). The definition what exactly constitutes DT was formulated somewhat in parallel among dystonia and tremor experts, and as such viewed DT as either a movement manifestation of dystonia (53), or as the co-occurrence of tremor and dystonia (7). Although DT as a concept has been adjusted several times (6, 7, 14, 53, 54), there is still no unambiguous definition (28), and a recent expert consensus advised the use of descriptive terminology when assessing a patient presenting with a combination of dystonia and tremor, rather than vague, hybrid and potentially imprecise diagnoses (8). It is no surprise and in fact sobering to reflect that our understanding of the underlying pathophysiology in both conditions is equally ambiguous (9, 55), for as long as we cannot agree on what consistently constitutes ET or DT, we will not be able to unravel its origin.

### 4.3 Tremor associated with dystonia differs from dystonic tremor

Although based on a single-center sub-analysis in a limited sized dataset, our preliminary comparison between ET, DT and TaD using un-biased, feature-based machine learning confirms the observation that DT and TaD differ substantially in their signal characteristics (42). Our findings are consistent: standard characteristics, feature-based analysis using the HCTSA Interface, as well as supervised machine learning results differ more between DT and TaD than ET and TaD. Although in line with previous reports (42), the replication of our observations in larger datasets is warranted.

### 4.4 Relevance for future tremor diagnostic criteria

The ultimate aim for the adequate differentiation between disorders, in addition to understanding the underlying pathophysiology, should be their optimal treatment. As such, every attempt to define diagnostic criteria should aim to stratify patients to guide treatment decisions. Current diagnostic criteria (or their implementation), apparently do not provide precise guidance for the reliable and replicable differentiation between ET and DT. Although we acknowledge the relevance of astute clinical phenotyping in routine practice, our results call for revisions to the current criteria, which do not take a) the underlying pathophysiology or b) therapeutic response into account (9). We are convinced that electrophysiology (3) and in particular accelerometry (13, 29) are helpful in the characterization of tremor syndromes. Indeed, there are first reports that tremor signal analysis allows therapeutic stratification: in both invasive (56) as well as non-invasive electrical brain stimulation studies (31, 57) more sinusoidal, i.e., regular tremor is much more likely to respond to electrical stimulation. To our knowledge, this has not been studied in other tremor interventions, but if replicated, would strongly argue to stratify tremor conditions according to therapeutic response and phenomenological characteristics.

### 4.5 Limitations

There are possible limitations to this work: our analyses are based on accelerometer signals only, whereas the clinical diagnosis takes additional clinical information into account. Even more, a diagnostic pipeline only based on the analysis of oscillatory information is unable to capture the postural/tonic domain of movements, which may hold the diagnostic essence of DT. Future studies therefore should include both domains. Many of the included original recordings were limited to 10–15 s length. Although preliminary analyses did not show an increase in classification accuracy in 15 s recordings, we cannot rule out that longer recording durations could improve classification accuracy. Unfortunately, independent external expert video ratings were not ascertainable, as video recordings were not available for all patients included. As ground truth in tremor diagnosis is nebulous at best, however, we are of the opinion that external expert ratings would not add relevant value, as they are similarly prone to subjective diagnostic bias (22). Although multi-centric, our sample contains a limited number of data points per diagnosis, and we recognize that a larger sample would be desirable, in particular when applying machine learning algorithms. At the same time, the variance to graph distributions calculated indicate an even representation of tremor diagnoses across centers, suggesting our sample to be representable of tremor phenotypes across these diagnostic categories. Ultimately, we chose a rather limited but well-curated data set from centers with dedicated tremor expertise, as outliers can significantly skew machine learning results in time-series data (29).

The comparison of data from different centers inherently poses difficulties in terms of signal compatibility due to clinical and technical inconsistencies. However, our approach to data curation and standardization has been particularly developed to integrate recordings from different centers (30). The resulting data inhomogeneity serves our aim to identify not clinician- and center- but disease-specific tremor characteristics from the resulting time-series data. It is therefore reassuring that findings from both standard and feature-based machine learning analyses converge on clearly identifiable key differences between the studied tremor entities.

## Data Availability

The raw data supporting the conclusions of this article will be made available by the authors, without undue reservation.

## Supplementary material

**Supplementary Table 1** Settings and positions of accelerometer specification of the accelerometer used for data collection and position at the hand.

**Supplementary Table 2** Standard tremor characteristics description of the tremor characteristics computed based on ^42.^

**Supplementary Table 3** Clinical and demographic details of participants.

**Supplementary Table 4** Clinical and demographic details of ET, DT and TaD participants from Graz.

**Supplement Table 5** Univariate classification accuracy of individual HCTSA Features in postural position between ET vs DT using Random Forest (RF) with 100 trees, bootstrapping and LOOCV.

**Supplementary Table 6** Supervised machine-learning based classification accuracy using the ideal combination of uncorrelated features. Despite the improvement over univariate classification accuracy for samples from each individual center with an individual classification accuracy of up to 90.2%, supervised machine learning algorithms showed only a max of 69.0% classification accuracy for the combined data set (in brackets number of uncorrelated features included).

**Supplementary Figure 1** Standard tremor characteristics across graz. distribution of AUC, peak frequency, TSI, half width power, full width at half maximum and peak power visualized using boxplots by diseases for DT, ET and TaD. FWHM showed statistical differences betweeen ET and TaD. AUC, TSI and Peak Power showed statistical differences between ET/DT and TaD^*^ < 0.05.
