# Supplementary material for: Signal-based differentiation of essential and dystonic tremor
Source: Front Neurol. 2026 Sep 7;17:1910178. doi: 10.3389/fneur.2026.1910178 (PMC13590316; doi:10.3389/fneur.2026.1910178)
Supplement: Supplementary file 1 [file Table_1.docx]

Supplementary Material

# 1 Supplementary Tables

|  | **Graz** | **Sevilla** | **London** |
| --- | --- | --- | --- |
| **Accelerometer** | Biometrics ACL300 + amplifier K800 | EGAS3 triaxial accelerometer | MMA7361, Freescale Semiconductor |
| **Position** | middle phalanx of index finger | middle phalanx of index finger | proximal phalanx middle finger |

**Supplemental table 1) Settings and positions of accelerometer**
Specification of the accelerometer used for data collection and position at the hand

| **Peak Frequency** | Frequency of highest peak in timeseries FFT |
| --- | --- |
| **Peak Power** | Power of highest peak in timeseries FFT |
| **Area under the curve (AUC)** | AUC of FFT |
| **Full Width at Half Maximum (FWHM)** | Frequency range of half peak frequency of FFT |
| **Half Width Power (HWP)** | Power range of half peak power of FFT |
| **Tremor Stability Index** | Tremor metric introduced by (Biase et al., 2017) |
| **Harmonics** | Harmonics with frequency f’ = n * peak frequency |

**Supplemental table 2) Standard Tremor Characteristics**
Description of the Tremor Characteristics computed based on (Panyakaew et al., 2020)

|  | **ET** |  |  |  | **DT** |  |  |  | **All** |  |  |
| --- | --- | --- | --- | --- | --- | --- | --- | --- | --- | --- | --- |
|  | **Graz** | **Sevilla** | **London** | **Comparison** | **Graz** | **Sevilla** | **London** | **Comparison** | **ET** | **DT** | **Comparison** |
|  | n=21 | n=10 | n=6 |  | n=9 | n=8 | n=10 |  | n=37 | n=27 |  |
| **Age** | 61.5±19.1 | 49.3 ± 17.7 | 54.5 ± 16.5 | P=0.21 | 63.9±14.0 | 62.3 ± 10.6 | 66.1 ± 14.9 | P=0.84 | 57.5±18.3 | 64.2 ±13.0 | P=0.12 |
| **Sex** | 5 f, 15 m  (1 missing) | 3 f, 6 m  (1 missing) | 3 f, 3 m | P=0.53 | 5 f, 4 m | 5 f, 3 m | 3 f, 6 m  (1 missing) | P=0.64 | 11 f, 24 m  (1 missing) | 13 f, 13 m  (1 missing) | P=0.11 |
| **Handedness (% right)** | 16r, 1 l  (4 missing) | 8 r, 1 l  (1 missing) | 6 r | P=0.70 | 5 r, 1 l  (3 missing) | 7 r, 1 l  87.50% | 6 r, 4 l  (2 missing) | P=0.83 | 30 r, 2 l  (5 missing) | 18 r, 4 l  (5 missing) | P=0.18 |
| **Age at onset (yrs)** | 40.6±21.7 | 30.6 ± 15.0 | 39.0 ± 17.2 | P=0.49 | 36.9±22.3 | 49.4 ± 10.9 | 36.6 ± 20.0 | P=0.31 | 37.8±19.4 | 40.8±18.8 | P=0.56 |
| **Disease duration (yrs)** | 20.0±21.7 | 16.3 ± 14.1 | 15.7 ± 10.3 | P=0.83 | 27.0±19.6 | 12.9 ± 9.4 | 31.6 ± 20.4 | P=0.10 | 18.2±18.0 | 24.2±18.6 | P=0.22 |
| f=female; m=male; r=right; l=left  **Supplementalal table 3) Clinical and demographic details of participants.** | | | | | | | | | | | |

|  | **ET** | **DT** | **TaD** |
| --- | --- | --- | --- |
|  | n=21 | n=9 | n=14 |
| **Age** | 61.5±19.1 | 63.9±14.0 | 63.6±10.4 |
| **Sex** | 15 m, 5 f  (1 missing) | 5 f , 4 m | 8f, 6 m |
| **Handedness (% right)** | 1 l, 16 r  (4 missing) | 1 l, 5 r (3 missing) | 12 R, 0 L (2 missing) |
| **Age at onset (yrs)** | 40.6±21.7 | 36.9±22.3 | 41.9±22.5 |
| **Disease duration (yrs)** | 20.0±21.7 | 27.0±19.6 | 21.6±19.8 |

**Supplemental table 4) Clinical and demographic details of ET, DT and TaD participants from Graz**

|  | **Graz** | **London** | **Sevilla** | **combined** |
| --- | --- | --- | --- | --- |
| **Maximum univariate classification accuracy** | 87.2% | 71.4% | 78.3% | 69.0% |
| **≥85%** | 3 | - | - | - |
| **≥80%** | 17 | - | - | - |
| **≥75%** | 109 | - | 7 | - |
| **≥70%** | 647 | 15 | 57 | - |
| **≥65%** | 1569 | 122 | 217 | 37 |
| **≥60%** | 2833 | 854 | 623 | 217 |

**Supplement table 5) Univariate classification accuracy of individual HCTSA Features in postural position between ET vs DT using Random Forest (RF) with 100 trees, bootstrapping and LOOCV**

|  | **Random Forest** | **Linear SVM** | **Neural Network** | **Linear Regression** | **k-nearest Neighbour** |
| --- | --- | --- | --- | --- | --- |
| **Graz** | 87.2% (1) | 89.6% (6) | 82.2% (4) | **90.2% (6)** | 83.3% (1) |
| **London** | **71.7% (8)** | 67.3% (6) | 61.1% (1) | 66.8% (7) | 71.4% (1) |
| **Sevilla** | **78.3% (1)** | 73.3% (8) | 65.9% (8) | 72.4% (5) | **78.3% (1)** |
| **Combined** | **69.0% (1)** | 66.3% (3) | 66.2% (1) | 66.9% (3) | 65.8% (3) |

**Supplemental table 6) Supervised machine-learning based classification accuracy using the ideal combination of uncorrelated features.**
Despite the improvement over univariate classification accuracy for samples from each individual centre with an individual classification accuracy of up to 90.2%, supervised machine learning algorithms showed only a max of 69.0% classification accuracy for the combined data set (in brackets number of uncorrelated features included).

# 2 Supplementary Figures

**Supplement figure 1) Standard Tremor Characteristics across Graz.**

Distribution of AUC, Peak Frequency, TSI, Half Width Power, Full Width at Half Maximum and Peak Power visualized using boxplots by diseases for DT, ET and TaD. FWHM showed statistical differences betweeen ET and TaD. AUC, TSI and Peak Power showed statistical differences between ET/DT and TaD*<0.05
